# Comparison of the Structure and Function of Phospholamban and the Arginine-14 Deficient Mutant Associated with Dilated Cardiomyopathy

**DOI:** 10.1371/journal.pone.0106746

**Published:** 2014-09-16

**Authors:** Eleri Hughes, David A. Middleton

**Affiliations:** Department of Chemistry, Lancaster University, Lancaster, United Kingdom; Tokyo Medical and Dental University, Japan

## Abstract

Phospholamban (PLB) is a pentameric protein that plays an important role in regulating cardiac contractility via a reversible inhibitory association with the sarcoplasmic reticulum Ca^2+^ATPase (SERCA), the enzyme responsible for maintaining correct calcium homeostasis. Here we study the functional and biophysical characteristics of a PLB mutant associated with human dilated cardiomyopathy (DCM), with a deletion of arginine at position 14 (PLBR14Δ). In agreement with recent findings, we find that PLBR14Δ has a reduced inhibitory effect on SERCA compared to wild type PLB (PLBWT) when reconstituted into lipid membranes. The mutation also leads to a large reduction in the protein kinase A-catalysed phosphorylation of Ser-16 in the cytoplasmic domain of PLBR14Δ. Measurements on SERCA co-reconstituted with an equimolar mixture of PLBWT and PLBR14Δ (representing the lethal heterozygous state associated with DCM) indicates that the loss-of-function mutation has a dominant effect on PLBWT functionality and phosphorylation capacity, suggesting that mixed PLBWT/PLBR14Δ pentamers are formed that have characteristics typical of the mutant protein. Structural and biophysical analysis of PLBR14Δ indicates that the mutation perturbs slightly the helical structure of the PLB cytoplasmic domain and reduces its affinity for the phospholipid bilayer surface, thereby altering the orientation of the cytoplasmic domain relative to the wild-type protein. These results indicate that the structure and function consequences of the R14 deletion have profound effects on the regulation of SERCA which may contribute to the aetiology of DCM.

## Introduction

Cardiac contractility is determined by tight regulation of calcium transients within cardiac myocytes, with the force of contraction and rates of relaxation in part determined by the calcium-pumping action of the sarcoplasmic reticulum Ca^2+^-ATPase (SERCA2a). Phospholamban (PLB) is a 52 amino acid single-pass transmembrane protein located in the sarcoplasmic reticulum (SR) membrane, where it exists in dynamic equilibrium between monomeric and pentameric forms [1], [2]. PLB acts as a critical regulator of SERCA2a, the action of which involves a finely-tuned balance between kinases and phosphatases that are essential for the regulation and control of PLB activity and in turn, regulation of SERCA [2]–[5].

Under conditions of low [Ca^2+^], the monomeric form of PLB interacts with SERCA reducing the calcium affinity of the enzyme and reducing the rate of calcium uptake by the sarcoplasmic reticulum (SR) [4]. Inhibition is relieved by an increase in [Ca^2+^] or by protein kinase A catalysed phosphorylation of PLB at Ser16 and/or Thr17 in response to β-adrenergic stimulation [6]. Phosphorylation of PLB is believed to alter the contacts between SERCA and PLB, relieving inhibition but not necessarily leading to dissociation of the two proteins [7]. The monomeric form of PLB appears to interact with SERCA [8], but pentamers may also be involved in the fine control of SERCA regulation by acting as a reservoir controlling the precise concentration of monomer available to interact with the enzyme [9], [10]. PLB comprises four structural domains: a helical cytoplasmic domain Ia (residues 1–16), a loop region (residues 17–22), a helical domain Ib at the membrane cytoplasmic interface (residues 23–30) and a helical transmembrane domain II (residues 31–52) [11]–[13]. The transmembrane domain lowers the affinity of SERCA for calcium at non-saturating concentrations and the cytoplasmic domain has a small effect on the maximal activity of SERCA, and contains the phosphorylation sites. The main structural state populated by PLB in cell membranes is believed to be a so-called T state, in which the cytoplasmic helices are oriented approximately perpendicular to the transmembrane helix and making contact with the surface of the lipid bilayer [14]. Phosphorylation increases the population of a dynamic, structurally disordered “R state” which detaches from the membrane [14], [15].

Mutations in human PLB are known to cause familial cardiomyopathies; in particular there have been identified two mutations that result in dilated cardiomyopathy (DCM), the variants PLB R9C (a substitution of residue Arg9 for Cys), and PLB R14Δ (a deletion of residue Arg14) [16]–[18]. The R9C mutation is a dominant loss-of-function (LOF) mutation, which generates a highly pentameric form of PLB as a result of the extra cysteine residue and, in consequence, less of the monomeric form is available to interact with SERCA [19], [20]. Monomeric R9C, which exerts an inhibitory effect on SERCA, is fully capable of being phosphorylated with subsequent relief of inhibition [19], but sequestration into the pentamer renders R9C unavailable for phosphorylation by cAMP-dependent protein kinase A (PKA), which cannot gain access to Ser16. The R14Δ mutation alters the signal sequence for correct location of PLB to the SR [21], and also disrupts the upstream consensus sequence for PKA phosphorylation at S16. Studies in mice reveal that in the absence of wild-type PLB (the homozygous state), R14Δ results in incorrect localisation to the plasma membrane allowing PLBR14Δ to interact with and activate Na^+^ K^+^-ATPase (NKA) [22]. This activation may be direct or indirect by interfering with the naturally inhibitory interaction between NKA and phospholemman (PLM). In the presence of equimolar PLBWT (the heterozygous state associated with DCM), correct localisation of PLBR14Δ to the SR occurs, allowing it to interact with and inhibit SERCA. Haghighi and co-workers showed that heterozygous PLB has a super-inhibitory effect on SERCA when co-expressed in HEK-293 cells, which is only partially reversed after phosphorylation of PLB [17]. Ceholski and co-workers subsequently reported that deletion of Arg14 disrupts the PKA recognition motif in the PLB cytoplasmic domain, which abolishes PLB phosphorylation and leads to constitutive inhibition of SERCA [23].

Here we study the structure-function relationship of PLB R14Δ reconstituted into phospholipid membranes with SERCA in the presence and absence of PLBWT. We show that the mutant in these membranes has a slightly reduced inhibitory effect in comparison to wild-type PLB and is resistant to phosphorylation by PKA. Structural analysis using solid-state nuclear magnetic resonance (ssNMR) and circular dichroism spectroscopy indicate that the functional differences are related to rather subtle alterations in the structure and topology of the protein.

## Materials and Methods

### Materials

Synthetic peptides PLB_1-23_WT and PLB_1-22_R14Δ, comprising the first 23 (WT) or 22 (R14Δ) cytoplasmic residues of PLB and N-terminally acetylated were supplied by Peptide Protein Research (PPR Ltd) (Fareham). Full-length synthetic PLBR14Δ was purchased from Activotec Ltd (Cambridge). PLB antibodies A1 and PS16 were purchased from Badrilla, and positive reactions visualised with the WesternBreeze chromogenic immunodetection kit from Invitrogen. Deuterated phospholipids were purchased from Avanti-Polar Lipids Inc, amylose resin from New England Biolabs (NEB), and all other chemicals obtained from Sigma. Microscope cover glass slides (8 mm×22 mm; thickness No. 0 (0.08 to 0.12 mm), used for preparation of oriented protein/lipid bilayers were purchased from Paul Marienfeld GmbH & Co. KG (Germany). SERCA1a Ca^2+^-ATPase was prepared from fast-twitch rabbit skeletal muscle according to a method adapted from East and Lee [24]. For assay purposes, purified SERCA1a membranes were reconstituted in unsaturated L-α-dioleoylphosphatidylcholine (DOPC) membranes alone or co-reconstituted with full-length PLB.

### SDS-PAGE

Analysis of the oligomeric state of PLB was carried out by SDS PAGE using the Bolt system (Life Technologies). Samples were prepared as per kit protocol and run on pre-cast 4–12% gradient Bolt Bis-Tris Plus gels using the MES SDS running buffer.

### Expression of PLBWT and PLBR14Δ

The PLBR14Δ expression construct was generated from the human PLBWT DNA sequence, using site-directed ligase-independent mutagenesis (SLIM) [25]. Full length (non-acetylated) PLBWT and the R14Δ mutant were expressed and purified using an adaptation of the procedure described by Buck et al. [26]. Both proteins were expressed in E. coli C41(DE3) as a fusion construct with maltose binding protein (MBP) using the pMAL c2X vector (New England Biolabs Inc.) containing the required PLB gene sequence preceded by an engineered TEV cleavage site [27], [28]. After purification on an amylose affinity column and cleavage with TEV protease [26], PLB was precipitated and isolated from MBP using a chloroform/methanol extraction procedure [29]. Briefly, the precipitate, containing PLB, was collected by centrifugation and dried. The sample was then re-suspended in 50/50 chloroform: acidified methanol (containing 1% acetic acid final concentration), and stirred at room temperature for two days. The sample was then filtered through a sinter-glass funnel under vacuum and the solution, containing solubilised PLB dried under N_2_ and high vacuum. Protein purity and identity was confirmed by MALDI-MS.

### Co-reconstitution of PLB with SERCA

PLB variants were reconstituted with SERCA in DOPC membranes at a lipid/PLB/SERCA ratio of 160∶10∶1 using an adaptation of methods described previously [30]–[32]. Reconstitution was carried out with SERCA1a, the abundant fast-twitch skeletal muscle form of the enzyme, which is well established as a model for SERCA2a function and its inhibition of PLB [33] and is widely used as a proxy for the cardiac enzyme in structural and biophysical characterizations. Briefly, DOPC or DOPC and PLB were dissolved in 50/50 chloroform/methanol and then dried to a film under a stream of N_2_ before leaving under high vacuum overnight. For samples containing PLB only, the dry films were resuspended in 10 mM phosphate buffer, pH 7.4. For co-reconstitutions with SERCA, the dry films were dissolved in OG buffer (10 mM Tris, 0.25 M sucrose 6 mg/ml octyl glucoside (OG); pH 7.5), prior to addition of SERCA membranes. Samples were allowed to stir at room temperature for 15 minutes and then at 4°C for 45 minutes. This was followed by 3 additions of 25 mg wet biobeads/hour with stirring, for a total of 3 hours. Membranes were removed from the biobeads, subjected to two freeze-thaw cycles and frozen at −20°C until ready for assay.

### SERCA activity measurements

ATPase activities of SERCA reconstituted alone, with PLBWT, with PLBR14Δ or with 1∶1 PLBWT: PLBR14Δ were measured in a final volume of 200 µl, using 5–10 µg SERCA1a reconstituted as described above. CaCl_2_ was added to the required free calcium concentration. Activity measurements were determined either by a rapid quench method, or by real time monitoring. For the quench method, samples were initially prepared in a total volume of 120 µl of reaction buffer (30 mM Tris, pH 8) to which 80 µl of assay medium (4 mM ATP, 4 mM MgCl_2_, 0.5 mM EGTA, 30 mM imidazole, pH 7.5) was added. The samples were incubated for 10 minutes at 37°C and the specific Ca^2+^-ATPase activity was quantified as the amount of inorganic phosphate (P_i_), liberated upon hydrolysis of ATP as measured by formation of a phospho-molybdate complex under acidic conditions [34]. Real time enzyme activity was determined on a Molecular Devices FlexStation3 plate reader using a standard coupled enzyme assay [35]. The measured activities were normalized to the maximum activity measured over the calcium concentration range and K_Ca_ values were calculated by non-linear least squares fitting of an equation for cooperative substrate activation (Hill analysis).

### Phosphorylation of PLB

Phosphorylation was carried out by an adaptation of a previous method, using 0.15 mg each of PLB and R14Δ solubilised in 0.3 ml of phosphorylation buffer (100 mM MOPS, 10 mM KCl, 8 mM MgCl_2_, 0.9% OG, 50 nM calyculin A; pH 7.1) [36]. 400 U of protein kinase A catalytic subunit (PKA), were dissolved in 800 µl 6 mg/ml dithiothreitol (DTT) in MilliQ grade water and left at room temperature for 10 minutes prior to use. 100 U (200 µl) were added to each sample to be phosphorylated including a PKA buffer-only control. Addition of 200 µl 6 mg/ml DTT was made to non-PKA controls. Phosphorylation was monitored in real time by a coupled enzyme assay [19], [37], with 165 µl of the appropriate phosphorylation reaction mixture added to PLBWT, PLBR14Δ, 1∶1 PLBWT:PLBR14Δ and buffer only control in a 96 well plate, together with a 30 µl volume containing phosphoenolpyruvate (2 mM), NADH (600 µM), pyruvate kinase (2 U), and lactate dehydrogenase (2 U), in 50 mM imidazole, 100 mM KCl, 8 mM MgCl_2_, 0.5 mM EGTA; pH 7.5. Phosphorylation was initiated with 5 µl of 100 mM ATP (pH 7), using the FlexStation 3 plate reader and monitored at A^340^ over several hours for the production of ADP, where [ADP]  =  ΔA^340^/6.22 mM^−1^cm^−1^. Initial rates of phosphorylation were calculated by linear regression analysis of the A_340_ values over the first 20 min after initiation of the reaction.

Successful phosphorylation was further determined by antibody dot blot analysis using anti-PLB antibody A1 and anti-phosphoPLB antibody PS16 (Badrilla). Equal volume aliquots of each protein or the mixture were applied to two PVDF membranes; these were then processed simultaneously using the same solutions differing only at the primary Ab step.

### Statistical analysis

K_Ca_ values and phosphorylation rates are presented as means ± SEM. Multiple group comparisons were performed using one-way ANOVA with Tukey's post hoc test (Minitab 17). P<0.05 was considered significant in all analyses.

### Spectroscopic measurements

Far-UV circular dichroism measurements were carried out on a Jasco J-180 spectropolarimeter. Circular dichroism (CD) spectra were recorded from 250 to 195 nm using a 0.1 cm cuvette, a scan rate of 20 nm/min and an average of 4 accumulations per sample. All peptides were prepared to 100 µM in 10 mM phosphate, pH 7. Measurements were made using a stepwise titration with trifluoroethanol (TFE) to generate measurements up to 40% TFE. Controls were carried out by titrating TFE into buffer minus the peptides and deducting the values obtained from the experimental values.

### Isothermal titration calorimetry

Heat flow resulting from binding of lipid small unilamellar vesicle (SUV's), to NAcPLB_23_WT or NAcPLB_23_R14Δ was measured using the ultrasensitive iTC_200_ MicroCalorimeter (MicroCal LLC, Northampton, MA). Reaction cell volume and total injection volume were 200 µl and 40 µl respectively. Experiments were performed at 25°C, at a power reference setting of 6 µcal/sec with stirring at 800 rpm. Data analysis was carried out using the Origin v.7 software (MicroCal) and fitted using the ‘one set of sites’ model. The reaction cell contained a 100 µM solution of peptide in 10 mM Tris; 1 mM EDTA, 30 mM NaCl; pH 7.4. Lipid SUV's comprising 10 mM 2∶1 L-α-dimyristoylphosphatidylcholine/L-α-phosphattidylglycerol (DMPC/DOPG) in the same buffer, were prepared using a probe sonicator and sonicated at 50% duty cycle for 2×30 s and injected via the syringe. Titrations were carried out at intervals up to 2 minutes in 1 or 2 µl aliquots following an initial 0.5 µl discard aliquot. Each injection generates a heat of reaction, determined by integration of the individual peaks from the heat flow trace.

### NMR measurements on oriented membrane samples

Uniformly ^15^N labelled full-length PLBWT or PLBR14Δ prepared recombinantly were reconstituted with 4∶1 DOPC/DOPE (L-α-dioleoylphosphatidylethanolamine) with a total of 50 mg of lipid. Samples were solubilised in up to 2 ml 50/50 chloroform: acidified methanol (1% acetic acid), before transferring to ∼ 25 glass plates (8×22 mm, thickness No. 0 (0.08–0.12 mm)), in a series 20 µl repeated aliquots, which were allowed to dry between additions. The plates were then transferred to a desiccating cabinet for several days and allowed to dry. The samples were hydrated either by applying a 10 µl aliquot of MilliQ grade water and transferring to a humidifier at 37°C or in the case of PLBR14Δ by omitting the addition of water and transferring directly to the humidifier. The samples were again left for several days until fully hydrated before stacking and wrapping in cling film ready for analysis by solid state NMR. The stacked plates were inserted into a Bruker double resonance flat coil probehead equipped with coil of dimensions 9×9×3 mm and ^15^N NMR spectra were acquired on a Bruker Avance 400 NMR spectrometer operating at a frequency of 400.13 MHz for protons. Spectra were obtained with Hartmann-Hahn cross polarization at a contact time of 1 ms and proton field of 63 kHz, and recycle delay of 1 s. Each spectrum was the result of accumulating between 300,000 and 400,000 transients, and so the experimental temperature was reduced to −10°C to avoid sample dehydration over the lengthy acquisition times. ^31^P spectra of the lipid headgroups were obtained under the same conditions before and after the ^15^N measurements to confirm the macroscopic alignment of the membrane sample.

### Magic-angle spinning NMR

Chain perdeuterated DMPC membranes containing PLBWT or PLBR14Δ (at a lipid:protein molar ratio of 20∶1) were rehydrated in 10 mM phosphate, pH 7 and centrifuged at 13000 rpm in a bench top microfuge. Pellets were transferred to a 4 mm diameter zirconia magic angle spinning (MAS) rotor. NMR measurements were carried out on a Bruker Avance 400 spectrometer equipped with a 4 mm triple resonance magic-angle spinning (MAS) probehead. The refocused INEPT (rINEPT) experiment was used to obtain two-dimensional ^1^H-^13^C spectra at 4°C and a MAS frequency of 8 kHz. Spectra were acquired using the States-TPPI method to obtain phase-sensitive quadrature detection in the indirect dimension and 64 increments were collected in t_1_.

## Results

### Effect of the R14Δ mutation on the PLB oligomeric state

**Figure 1:**
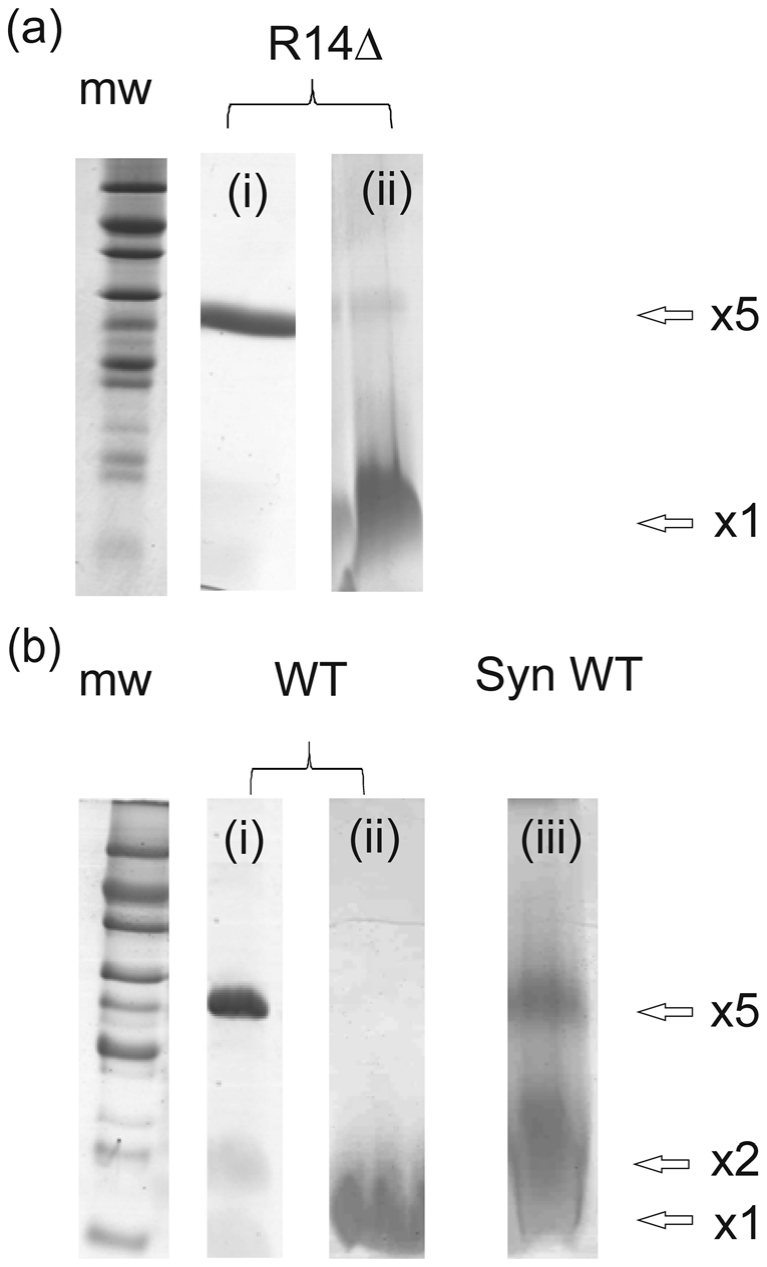
Analysis of the oligomeric distribution of wild-type and mutant PLB by SDS-PAGE: Comparison of recombinant PLBWT and PLBR14Δ in isolation and reconstituted with SERCA (Rec WT and Rec R14 Δ) at an approximate molar ratio of 20:1 PLB/enzyme. SERCA is also shown alone (right hand lane). All samples were prepared following the standard protocol and solutions provided with the Bolt system (Life Technologies), and then stained with standard Coomassie Brilliant Blue R250. The SeeBlue Plus2 Pre-Stained Standards were also supplied with the Bolt system; the apparent molecular weights shown on the gel are those expected when run on a NuPAGE Novex 4–12% Bis-Tris Gel with MES. Protein migration patterns are dependent on the buffer system used; in a more typical Tris/Glycine system, the migration pattern of the same markers would differ, with apparent molecular weights as follows: 4, 6, 16, 22, 36, 50, 64, 98, 148 and 250.

Immunoblotting analysis of N-flag tagged PLB suggested that the R14Δ mutation may partially destabilise the pentamer form of the peptide generating different sized oligomers [17]. To investigate the oligomeric state of untagged mutant and wild-type PLB proteins in SDS detergent (Figure 1), samples of recombinant PLBWT and PLBR14Δ both in isolation and co-reconstituted with SERCA under the same conditions as those used for functional assays, were solubilised in lithium dodecyl sulphate (LDS) sample buffer and analysed by SDS-PAGE. Both recombinant variants in isolation show a high proportion of pentameric protein (lanes 2 and 5). In the presence of SERCA, PLBR14Δ (lane 6), is still predominantly pentameric with a small amount of monomeric/dimeric protein present, and PLBWT (lane 3), demonstrates a greater shift towards the dimeric and monomeric forms. Hence in our hands, untagged PLBR14Δ retains a high propensity to assemble into pentamers in a lipid-like environment. However, these data do not rule out the possibility that subtle differences in the pentamer-monomer distributions of PLBWT and PLBR14Δ could occur in more native-like membrane environments, which might alter their relative effects on SERCA function.

### Effect of the R14Δ mutation on PLB function

**Figure 2:**
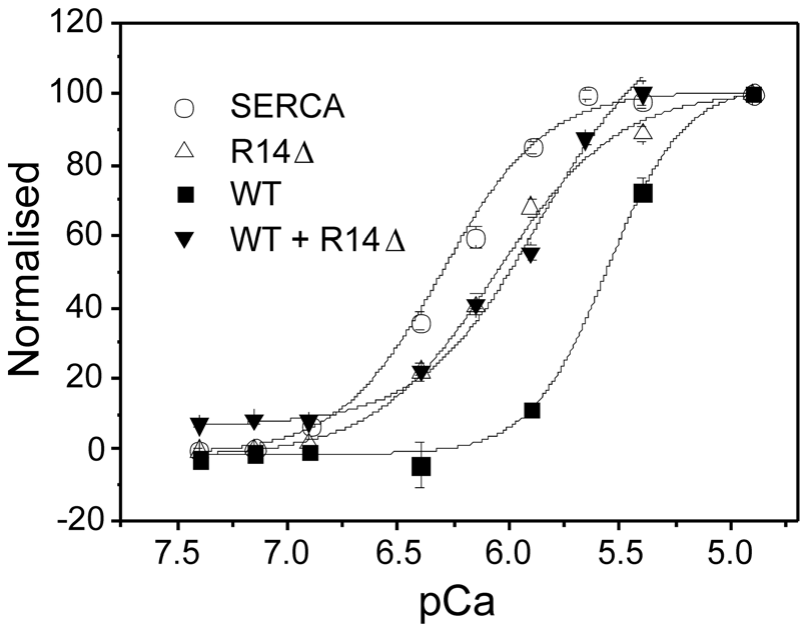
ATPase activity of SERCA: Normalised ATP hydrolysis by SERCA alone (open circles), or reconstituted with full-length recombinant PLBWT (squares), PLBR14Δ (open triangles), or an equimolar mix of mutant and WT (inverted triangles), with activity measured as a function of calcium concentration (n = 6). Samples were reconstituted with the enzyme in DOPC membranes at a lipid/protein/enzyme ratio of 160:2:1 and activities were determined in real time using the coupled enzyme assay.

Studies were carried out to determine the regulatory effect of recombinant PLBWT, PLBR14Δ and an equimolar mixture of the two (i.e., analogous to the heterozygous state) on SERCA when incorporated with the enzyme in DOPC membranes. Calcium-dependent ATPase activity measurements (Figure 2 and Table 1) confirm that PLBWT significantly lowers the affinity of SERCA for calcium relative to the control (SERCA only) sample. PLBR14Δ also lowers the calcium affinity of SERCA relative to the control, but is significantly less inhibitory than PLBWT under the same conditions (Table 1). This trend agrees with Ceholski et al. [23], who also reported a slightly reduced inhibitory effect of PLBR14Δ alone compared to PLBWT. In an equimolar mixture of PLBWT and PLBR14Δ the inhibitory effect is not significantly different from that of the mutant form alone (Table 1), in agreement with the findings of Ceholski et al [38]. As argued by these authors, the lack of the superinhibitory effect reported by Haghighi et al. [17] may be a result of separating the proteins from other cellular components into proteoliposomes.

**Table 1:** Activity of SERCA when reconstituted with PLB variants.

	k_Ca_	Δpk_Ca_
Control (SERCA only)	0.50 (±0.30)^a^	-
With PLBWT	2.75 (±0.42)^b^*	−0.74
With PLBR14Δ	0.89 (±0.10)^c^	−0.25
With 1:1 PLBWT:PLBR14D	0.70 (±0.15)^c^	−0.15

Values are means ± SEM. Ca^2+^-ATPase activities of reconstituted samples (n = 6) were measured over calcium concentrations from pCa 7.5 to pCa 5.0. Kca is the calcium concentration (in µM) required for half-maximal activity, determined by Hill analysis. pk_Ca_ is the negative logarithm of k_Ca_. Means that do not share a letter (superscript a, b or c) are significantly different (P<0.05). *indicates p<0.01 vs control.

**Figure 3:**
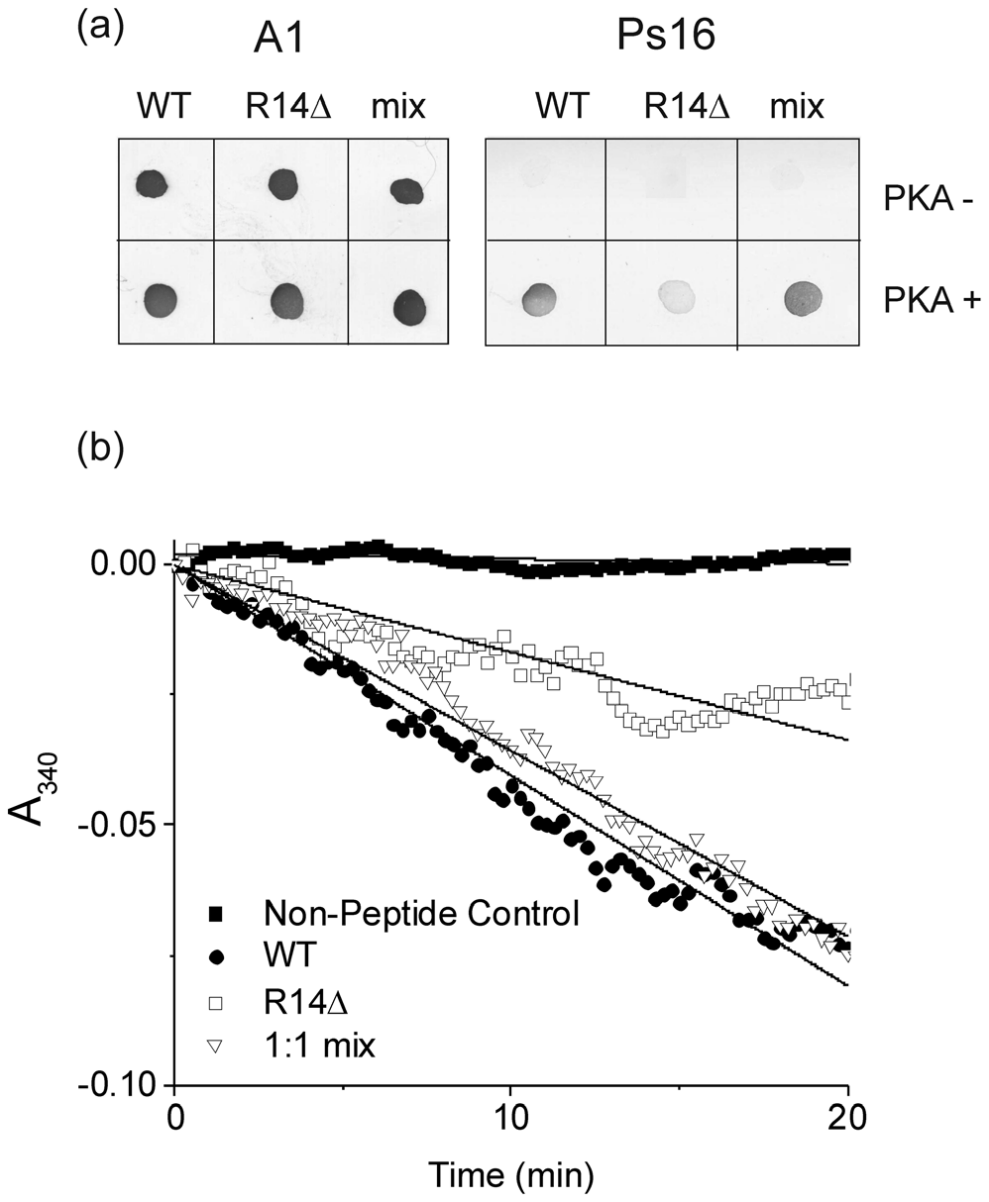
PKA-catalysed phosphorylation of PLBWT and PLBR14Δ : Relative ability of PKA to phosphorylate PLBWT, PLBR14Δ and an equimolar mix of PLB conformers was measured by a) dot blot analysis using PLB Ab A1 to detect both phosphorylated and unphosphorylated protein and Ab PS16 to detect phosphorylated forms of PLB only; b) real time measurement of ADP production resulting from successful PKA phosphorylation. Data (n = 3) were measured in triplicate in a single 96 well plate experiment and for clarity only the mean values are shown.

PLBR14Δ was next tested for its ability to undergo PKA-catalysed phosphorylation when incorporated alone into DOPC membranes and whether it interferes with phosphorylation of PLBWT in a 1∶1 mixture. The reaction was carried out with protein at a concentration of ∼40 µM and the presence of phosphorylated protein was confirmed by antibody (Ab) dot blot (Figure 3a). Ab A1 reacts with all forms of PLB, generating a positive control for the presence of PLBWT and PLBR14Δ, whereas Ab PS16 reacts only with phosphorylated forms of PLB. Figure 3a indicates that PLBWT and PLBR14Δ stain equally strongly with A1, but PLBR14Δ stains substantially more weakly with PS16 than does PLBWT, suggesting that the R14 deletion impairs phosphorylation. A negative control, comprising the first 26 residues of sarcolipin (SLN_1-26_), which is highly homologous to PLB, generated no reaction with either PLB Ab (data not shown). It should be noted that the R14Δ mutation occurs within the epitope region for A1 (L^7^ – S^16^), and PS16 (R^9^ – S^16^ (PO_4_)), and could therefore negatively impact on their ability to react to the mutant form of PLB. The PS16 Ab is more likely to be affected since this is much more specific than A1, a more promiscuous Ab capable of reacting with multiple forms of PLB. This may in part account for the minimal reaction of PS16 with PKA treated PLBR14Δ. Qualitative inspection of the PS16 blot for the mixed PLBWT and PLBR14Δ sample in Figure 3a implies that PLBR14Δ does not substantially impair the ability of PKA to phosphorylate PLBWT.

While Ab dot blot analysis is a useful technique for detecting and confirming the presence of phosphorylated protein, it tells us nothing about the rate or efficiency of phosphorylation. A second measure of phosphorylation was used, therefore, in which the production of ADP during the PKA-catalysed reaction is linked to oxidation of NADH using the coupled enzyme assay, and monitored by observing the absorbance at A_340_ (Figure 3b). This allows PKA activity to be monitored in real-time (Figure 3b) [37], and is a technique used in previous studies with the PLBR9C mutant [19]. Measurements were carried out for both PKA treated samples and untreated controls in addition to a PKA control in the absence of PLB protein. The rate of NADH oxidation is determined by the concentration of ADP present in the samples, which in turn is determined by the level of successful phosphorylation. Figure 3b clearly shows that phosphorylation of PLBR14Δ alone occurs at a significantly slower rate (16±2 µM h^−1^) than the phosphorylation of PLBWT (38±3 µM h^−1^, p<0.05), as reflected in the dot blotting data representing the end-point of the phosphorylation reaction. In an equimolar PLBR14Δ/PLBWT mixture the overall rate of phosphorylation of PLBWT (30±4 µM h^−1^) is not significantly different from PLBWT alone (p>0.05), also in agreement with the dot blotting data. Together these results indicate that the R14Δ mutation impedes PKA-dependent phosphorylation of PLBWT, as reported in earlier work [23].

### Structure and dynamics of the PLBR14Δ cytoplasmic domain

**Figure 4:**
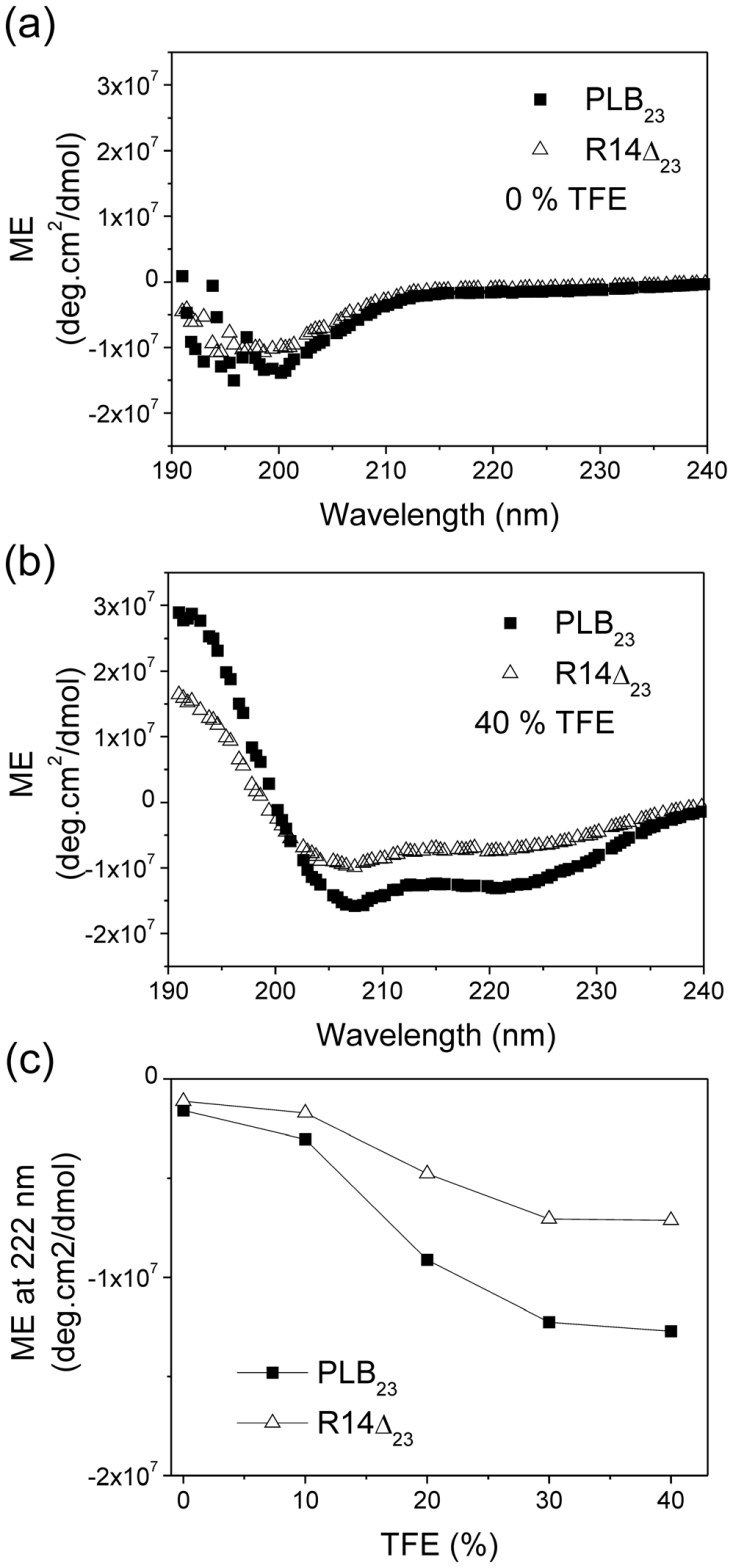
Secondary structure comparison of the cytoplasmic domains (residues 1-23) of PLBWT and PLBR14Δ by circular dichroism : Spectra are shown for peptides dissolved in a) water and b) 60% water:40% TFE. c) Molar elipticity at 222 nm monitored during TFE titration from 0% to 40% to determine the peptides relative ability to form secondary structure. A decrease in molar elipticity at 222 nm is diagnostic of a shift towards an α-helical structure.

We next investigated whether the functional characteristics of the R14Δ mutation, and its resistance to phosphorylation in the cytoplasmic domain, originate from structural modifications to the protein. Peptides representing the cytoplasmic domains of PLBWT and PLBR14Δ (PLB_1-23_WT and PLB_1-22_R14Δ) were analysed for differences in overall secondary structure using CD spectroscopy and titrating the peptides with TFE to stabilise any intrinsic helical structure. The spectra are consistent with a shift from an unstructured state in 0% TFE (Figure 4a), to a more typically α-helical structure at 40% TFE (Figure 4b). Further titration of TFE into both peptide solutions did not result in further changes to the spectrum. By monitoring the decrease in molar ellipticity at 222 nm (after adjusting for sample volume), the spectra indicate that the WT sequence has a greater propensity to form an α-helix than the R14Δ mutant (Figure 4c).

**Figure 5:**
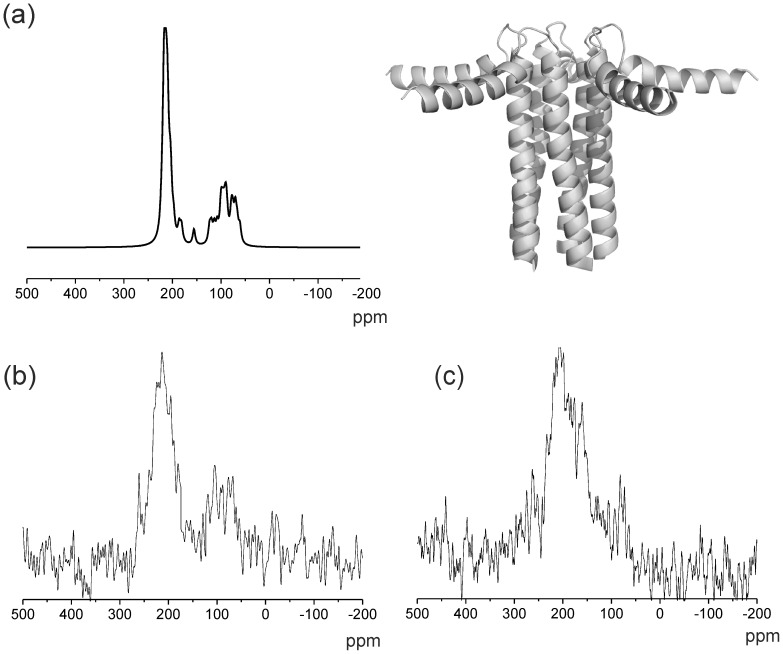
NMR analysis of the domain orientations of PLBWT and PLBR14Δ in aligned lipid membranes: ^15^N NMR measurements were carried out on aligned membranes of 4:1 DOPC/DOPE containing uniformly ^15^N labelled recombinant PLBWT or PLBR14Δ. a) “Pinwheel” structure of PLB in the T-state taken from the PDB coordinates (2KYV) with a simulated spectrum calculated from 2KYV. Only main-chain ^15^N resonances were included in the simulations of the spectra because of uncertainties in the orientation of the side-chains. b) Experimental spectrum for PLBWT. c) Experimental spectrum for PLBR14Δ.

We next turned attention to the effect of the R14 deletion on the membrane topology of PLB, and specifically the relative orientations of the cytoplasmic and transmembrane domains. We prepared uniaxially aligned samples of uniformly ^15^N labelled PLBR14Δ and PLBWT in DOPC/DOPE membranes and obtained ^15^N cross-polarization NMR spectra of the samples aligned in the magnet with the membrane normal approximately parallel with the applied field (as confirmed by the narrow ^31^P NMR spectra for the lipid headgroups; data not shown). Under these conditions the ^15^N spectra are consistent with the proteins having a unique orientation in the magnetic field, and despite the low signal to noise some differences between the spectra of wild-type and mutant proteins could be discerned. The spectrum of PLBWT exhibits a line shape with enhanced intensity around 60–120 ppm and 200 ppm and very little signal from 120–200 ppm. This line shape is consistent with helical domains oriented approximately perpendicular and parallel to the bilayer normal, respectively. A theoretical ^15^N spectrum was simulated from the atomic coordinates for PLBWT pentamers in the “pin-wheel” structure of the T-state (2KYV: Figure 5a) with the cytoplasmic domain approximately parallel with the membrane surface (12). The theoretical ^15^N spectrum is qualitatively rather similar to the experimental spectrum, suggesting that under these conditions (and in the same membrane composition as for the published structure) PLBWT adopts a structure reminiscent of the T-state. The spectrum of PLBR14Δ covers the 100–200 ppm range with a maximum around 200 ppm, suggesting that there is overall much more orientational disorder in the mutant (Figure 5b).

**Figure 6:**
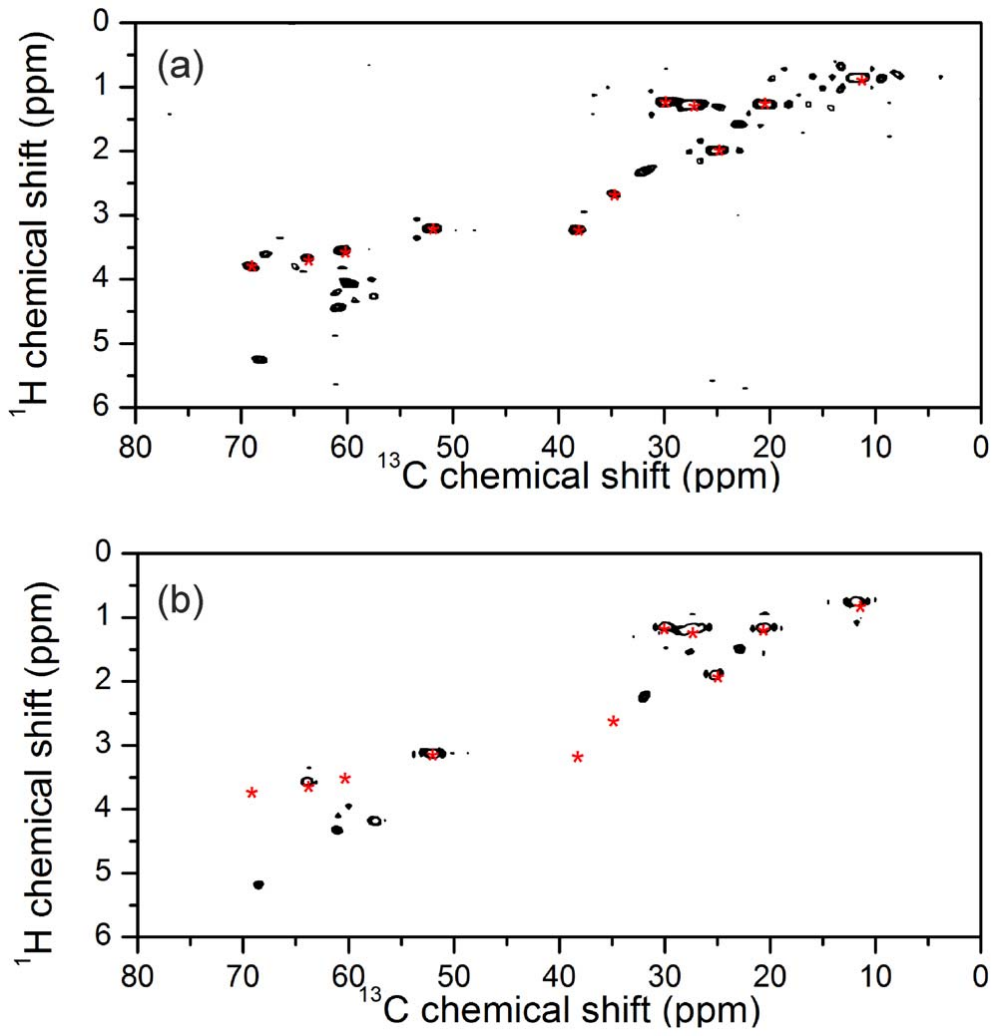
Solid-state NMR spectra of PLBWT and PLBR14Δ in unoriented membranes: ^1^H-^13^C rINEPT solid-state NMR spectra are shown for uniformly ^13^C-labelled PLBR14Δ (a) and PLBWT (b) prepared in 4:1 DOPC/DOPE membranes. Red asterisks indicate the positions of lipid resonances.

These results suggest that the R14 deletion tends to alter the average orientation of the cytoplasmic domain, such that it moves away from the membrane surface. PLBWT has been found to populate two major topological states in equilibrium, one in which the amphipathic cytoplasmic domain is structurally ordered and approximately parallel to the membrane surface (“T-state”) and the other in which the cytoplasmic domain is dynamically disordered and dissociated from the membrane surface (“R-state”) [14]. Here, a ^13^C-detected refocused INEPT magic-angle spinning NMR experiment was carried out to detect differences in the mobility of the cytoplasmic regions of uniformly ^13^C-labelled PLBWT and PLBR14Δ reconstituted into chain-perdeuterated DMPC membranes. The refocused INEPT experiment has previously been shown to detect the cytoplasmic region in the R-state because the mobility of this region gives rise to long transverse relaxation times [39]. Hence we reasoned that differences in the number and intensity of the observed resonances in the refocused INEPT spectra of the two PLB variants, all other considerations being equal, would signify differences in the mobility of the cytoplasmic domains and in the position of the equilibrium between the R- and T-states. The spectra of PLBWT and PLBR14Δ in DMPC membranes at 30°C are shown in Figure 6. The hydrocarbon chains of the lipid would normally give rise to a strong background signal and so DMPC was used here because the perdeuterated lipid is commercially available. Although it was not possible to assign the spectra from the measurements performed, it is clear that the spectrum of PLBR14Δ (Figure 6a) exhibits considerably more resonances than the spectrum for PLBWT (Figure 6b) owing to the longer ^1^H and ^13^C transverse relaxation times for the mutant. Hence the spectrum of PLBR14Δ suggests that the dynamic equilibrium is shifted toward a more disordered state in comparison to the more structurally ordered cytoplasmic domain of PLBWT.

**Figure 7:**
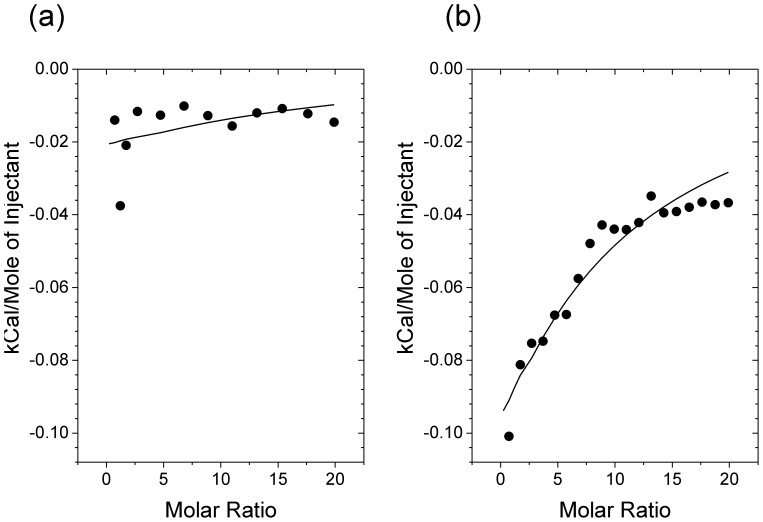
Analysis of PLB interactions with lipid SUV's using isothermal titration calorimetry: Cytoplasmic peptides PLB_23_R14Δ (a), and PLB_23_ (b), were titrated with 2:1 DMPC/DOPG lipid SUV's to determine their relative ability to associate with lipid membrane surfaces. Titration curves of integrated enthalpy versus mole ratio of reactants are shown with solid lines representing the best fitting curves.

The ordered T-state of PLB may depend in part on the amphipathic cytoplasmic helix being stabilised by electrostatic interactions with the membrane surface, i.e., the phospholipid headgroups. If PLBR14Δ is predisposed toward the more disordered state as suggested by the NMR measurements, this may arise from a weakening of the stabilising membrane interactions resulting from the loss of the cationic arginine residue. Isothermal titration calorimetry was used therefore to determine the relative affinities of the PLBWT and PLBR14Δ cytoplasmic domains (represented by PLB_1-23_WT and PLB_1-22_R14Δ) for phospholipid membrane surfaces. Previous work demonstrated that SUV's of 2∶1 DMPC/DOPG are a useful model system to compare the membrane affinities of amphipathic peptides, giving measureable enthalpic changes for PLB_1-23_WT ([40] and Figure 7a). Here, titrations of DMPC/DOPG SUV's into a solution of PLB_23_R14Δ do not give rise to measureable enthalpic changes (Figure 7b), indicating that for this combination of phospholipids the membrane interactions of PLB_23_R14Δ are substantially weaker than for PLBWT.

## Discussion

We have used a variety of techniques to investigate the functional and conformational properties of PLBWT and the mutant PLBR14Δ lacking R14 in the cytoplasmic domain. It is generally accepted that PLBWT reduces the K_m_ of SERCA for calcium, but it has been variably shown to decrease or increase V_max_ or to have no effect [32], [35], [41]–[44]. Here we find that PLBR14Δ is a slight loss of function mutant that is capable of interacting with SERCA and lowering its affinity for calcium, but to a lesser degree than PLBWT alone (Figure 2). When the mutant was reconstituted together with PLBWT, the functional effects are typical of those seen for PLBR14Δ alone. Previously, in work using HEK-293 cells transfected with N-Flag tagged PLB constructs, the R14Δ mutation had been described as super-inhibitory, but only when it occurs in the WT/mutant heterozygous state [17]. Consistent with our work they also found homozygous PLBR14Δ to be a slight loss of function mutation. Variation in results could potentially be explained by differences in both assay method and the PLB constructs used. Our results however agree with more recent work that examined a series of targeted mutations in the PLB cytoplasmic domain, and suggested that correct hydrophobic balance in this region is critical for correct regulation of SERCA [38]. It also highlights the importance of the role played by the cytoplasmic domain in the functional regulation of SERCA. The dominant effect of PLBR14Δ over PLBWT suggests that the mutant form successfully competes with the wild-type protein for association with the enzyme. This is also consistent with work seen elsewhere in which loss of function PLB mutants have been shown to compete with PLBWT for SERCA association in both reconstituted systems [45] and living cells [46].

The diminished capacity of PLBR14Δ to inhibit SERCA may arise from changes to the oligomeric status of the PLB mutant. It is widely recognised that PLB is more inhibitory as a monomer [8], [47], [48], than it is in its pentameric state and thus the reduced efficiency of SERCA inhibition by PLBR14Δ might reflect alterations in the monomer-pentamer equilibrium. It has been suggested that, in comparison to WT PLB, PLBR14Δ tends to be more monomeric [17] which was proposed to account for the super-inhibitory effect of the mutant observed in that study. Here, SDS-PAGE analysis indicates that while recombinant PLBWT and PLBR14Δ in isolation are both predominantly pentameric in detergent, when reconstituted with SERCA, PLBWT more readily dissociates in to the monomeric form than PLBR14Δ. In the absence of precise quantitative information on the populations of monomer and pentamer in lipid membranes, it cannot be ruled out that small differences in the monomer-pentamer ratios of PLBR14Δ and PLBWT give rise to the functional differences observed here.

The loss of function of PLBR14Δ may arise from the preference of the cytoplasmic domain to adopt a structurally disordered, membrane-disordered state akin to the R-state observed for the wild-type protein. The PLBWT cytoplasmic domain can undergo a shift from a folded, membrane-associated T-state to an unfolded, excited, membrane free R-state [10], [12], [14], [40]. CD analysis (Figure 4) indicates that the cytoplasmic domain of PLBR14Δ in aqueous solution has a lower α-helical content than the corresponding region of the wild-type protein. Moreover, NMR measurements (Figures 5 and 6) are broadly consistent with the cytoplasmic domain of PLBR14Δ being more dynamically disordered and oriented further away from the membrane surface compared to PLBWT. Reduced interactions with the membrane surface are supported by ITC measurements (Figure 7) which indicate that PLB_1-23_R14Δ interacts more weakly with membranes than does PLB_1-23_WT. The effects of the membrane environment on PLB structure and function are well established, with membranes representative of SR favouring a non-extended PLB conformation with an T-type structure [49]. In dodecylphosphocholine (DPC), the T (folded, membrane associated), and T'-states (partially folded, membrane associated), are observed whereas in DMPC the R-state (unfolded, membrane dissociated), predominates. Lipid head-group charge, bilayer curvature/thickness all influence the conformational equilibrium and the extent of PLB aggregation [14]. Molecular dynamics simulations of the PLB structure in DOPC bilayers [10], [12] revealed that residue R13 residues is solvent exposed and accessible to PLB binding partners, while R14 appears to dip in to the membrane surface contacting lipid bilayer head-groups where it forms hydrogen bonds with the oxygen atoms of the DOPC glycerol moiety. Hence deletion of R14 may weaken this interaction and destabilise the membrane-associated state of the mutant.

The phosphorylation measurements reported here (Figure 3) agree with earlier results [22], [23] and indicate that the R14 deletion impairs the PKA-dependent phosphorylation of PLB, either by modifying the enzyme recognition site, perturbing the protein conformation, or both. The lower inhibitory function of PLBR14Δ may thus be compensated by the resistance of the mutant to phosphorylation, which in the cellular environment may give rise to constitutive inhibition of SERCA and the disruption of calcium transients. Such effects in the long term may explain the occurrence of DCM in patients that possess the R14Δ mutation.
